# Case Report: Successful avoidance of etoposide for primary hemophagocytic lymphohistiocytosis-induced multiple organ dysfunction syndrome using emapalumab

**DOI:** 10.3389/fped.2023.1340360

**Published:** 2024-01-18

**Authors:** Timothy J. Hahn, Daniel J. McKeone, James W. Beal, Jessica E. Ericson, E. Scott Halstead

**Affiliations:** ^1^ Division of Pediatric Rheumatology, Department of Pediatrics, Penn State University College of Medicine, Hershey, PA, United States; ^2^ Dysregulated Immune Response Team (DIRT), Department of Pediatrics, Penn State University College of Medicine, Hershey, PA, United States; ^3^ Division of Pediatric Hematology and Oncology, Department of Pediatrics, Penn State University College of Medicine, Hershey, PA, United States; ^4^ Division of Pediatric Critical Care Medicine, Department of Pediatrics, Penn State University College of Medicine, Hershey, PA, United States; ^5^ Division of Pediatric Infectious Disease, Department of Pediatrics, Penn State University College of Medicine, Hershey, PA, United States

**Keywords:** MODS, sepsis, emapalumab, HLH, ferritin

## Abstract

We describe the case of an infant who presented with simple rhinovirus/enterovirus bronchiolitis whose condition worsened with rapid progression to multiple organ dysfunction syndrome (MODS). The patient was presumed to have either primary or secondary hemophagocytic lymphohistiocytosis (HLH), and treatment was initiated using dexamethasone, anakinra, and intravenous immunoglobulin to modulate the immune system. Due to the organ dysfunction, the use of etoposide was avoided and instead, emapalumab, an interferon gamma antagonist, was administered at a dose of 6 mg/kg. The patient's organ failure improved, and the levels of inflammatory markers decreased. The flow cytometry analysis revealed that cytotoxic cells lacked perforin expression, and subsequent genetic analysis confirmed homozygous pathogenic mutations in the perforin gene. This case highlights the potential avoidance of etoposide in cases of primary HLH, the possible benefit of an elevated initial dose of emapalumab, and the contribution offered by a multi-specialty team approach to complex diagnosis.

## Case report

**Figure 1:**
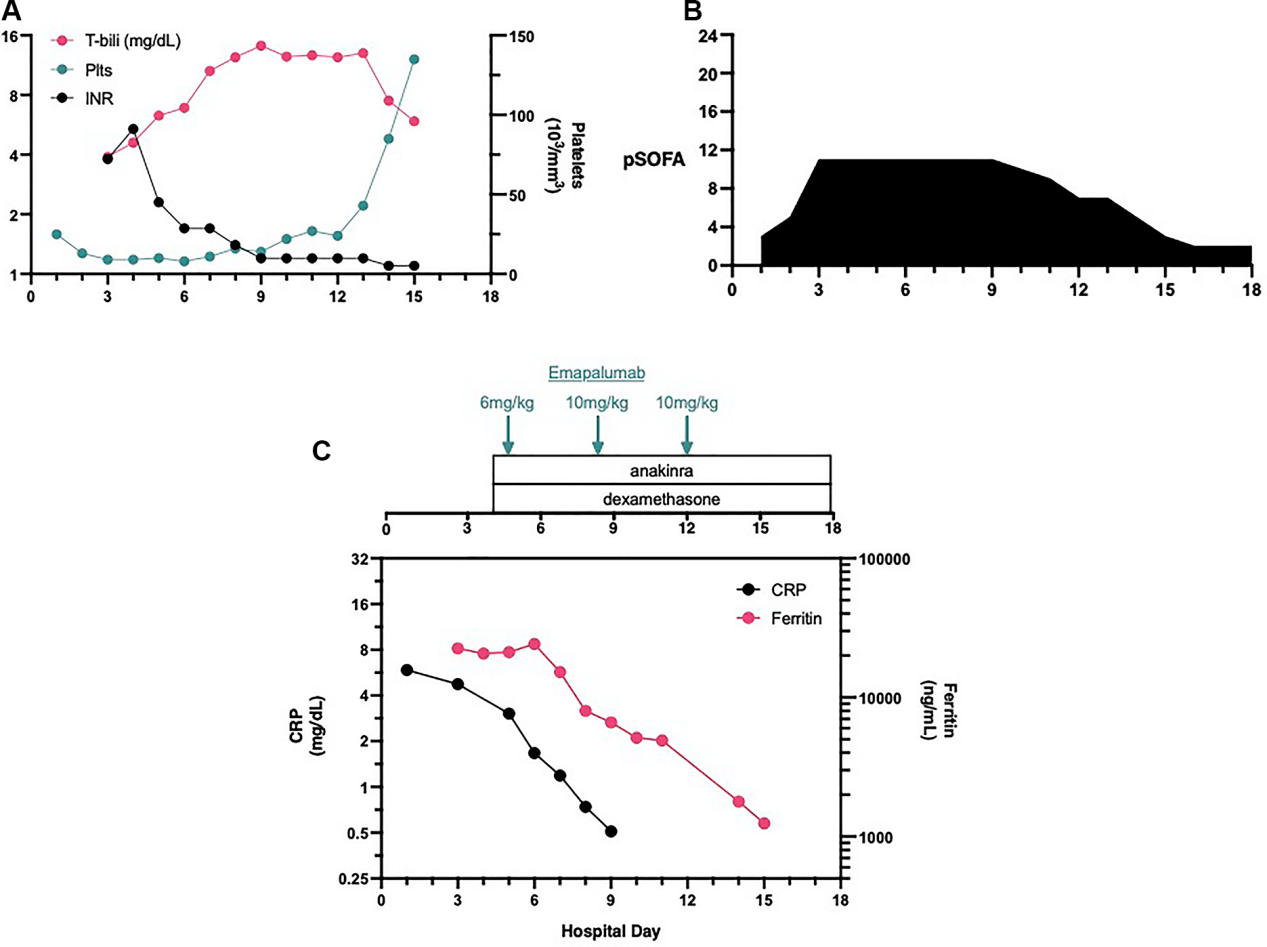
Laboratory values, organ dysfunction and interventions. The patient demonostrated thromobocytopenia on admission (**A**). When organ dysfunctioned worsened on HD#3 (**B**) additional laboratory values demonstrated coagulopathy and hyperbilirubinemia. Immune suppressive therapies were initiated late on HD#3 and the first dose of emapalumab was administered on HD#4 (**C**).

The patient is a 6-week old female infant, previously healthy, without any pertinent family history of the condition, who was admitted to Penn State Health Children's Hospital due to rhinovirus/enterovirus bronchiolitis and presented with a fever of 38.4°C. On hospital day (HD) #3, the patient was upgraded to the pediatric intensive care unit (PICU) for shock. Upon arriving at the PICU, the patient was intubated, and a right internal jugular central venous catheter was inserted. The laboratory tests demonstrated a condition of mixed acidosis (pH 7.15, PCO_2_ 53 mmHg, base deficit −20). The complete metabolic panel revealed the presence of hyponatremia (Na 122 mEq/L), and the complete blood count (CBC) demonstrated thrombocytopenia (platelets 13 × 10^3^ µl) (Figure 1A), a white blood cell (WBC) count of 26.53 × 10^3^ µl, and mild anemia with a hemoglobin level of 9.6 g/dl. The patient also demonstrated an elevated international normalized ratio (INR) and total bilirubin (Figure 1A) and hypofibrinogenemia (<60 mg/dl). The inflammatory biomarkers demonstrated an elevated ferritin level (16,995 ng/ml) and C-reactive protein (CRP, 4.25 mg/dl). While the liver and spleen were palpable on the physical exam, the liver ultrasound reported only “top normal liver and spleen size(s).” The patient demonstrated a pediatric sequential organ failure (pSOFA) score of 11 (3—respiratory, 4—coagulation, 1—cardiovascular, 1—renal, 2—hepatic) (Figure 1B) (1).

The Penn State University Department of Pediatrics recently assembled the Dysregulated Immune Response Team (DIRT), a group of pediatric subspecialists interested in hyperinflammatory disease processes. The team, comprised of Pediatric subspecialists from the Divisions of Pediatric Rheumatology, Pediatric Hematology/Oncology, Pediatric Infectious Disease, and Pediatric Critical Care offered differential diagnoses including viral (e.g., herpes simplex virus) or bacterial sepsis, primary and secondary HLH, and rickettsial infections. As of HD#3, the patient fulfilled four to five (depending on the interpretation of the abdominal ultrasound) of the HLH-2004 criteria (fever, cytopenias, hypofibrinogenemia, hyperferritinemia, and hepatosplenomegaly) (2). Given the similarity of the case to two previous documented cases of secondary HLH at our institution (3, 4), the diagnosis of HLH was presumed, and empiric treatment with dexamethasone and the IL-1 receptor antagonist, anakinra, was initiated. Meanwhile, an expedited order was placed for the interferon gamma antagonist, emapalumab (24 h transit time). Additional labs to confirm HLH were sent to the Diagnostic Immunology Lab at the Cincinnati Children's Hospital Medical Center (CCHMC).

We administered a moderate/high starting dose of emapalumab (6 mg/kg) very early in the morning on HD#5. Even prior to the administration of the second dose of emapalumab, the ferritin levels started to decline, indicating a decrease in macrophage activation (Figure 1C). However, organ failure persisted so the DIRT team recommended administering a higher dosage of emapalumab (10 mg/kg, maximal dose) for refractory disease. On HD#8, the patient’s organ functions showed improvement. The patient was extubated on HD#11 and transferred out of the PICU on HD#13.

The reference laboratory results were obtained from CCHMC around the time of extubation. On HD#9, the soluble CD25 (IL-2RA) level of 26,020 IU/ml was reported, and on HD#10, CXCL9 and IL-18 were reported at 108,813 pg/ml and 12,941 pg/ml, respectively. A bone marrow biopsy was performed on HD#11 once the coagulopathy had improved, revealing the presence of hemophagocytosis without the presence of malignancy. Finally, the flow cytometry results were received from CCHMC on HD#13 and demonstrated the absence of perforin expression by cytotoxic cells and NK cytotoxicity was absent, thus confirming a form of familial HLH. On HD#21, the genetic analysis demonstrated the presence of homozygous pathogenic early termination variants in *PRF1*, c.1122G>A (p.Trp374*). This finding confirmed our suspicion of primary HLH, specifically familial HLH type 2 (FHL2) (5).

## Discussion

This case highlights several interesting points pertaining to the diagnosis and treatment of HLH. MODS from sepsis/HLH/MAS is an end-stage disease process that can have many different infectious and rheumatologic etiologies (6, 7). Given the clinical overlap of these etiologies, we at Penn State Children's Hospital created the DIRT to help decipher the complex picture using a multi-specialty approach. At presentation on HD#3, we were fairly convinced that the patient, similar to a previously published case report (3, 4), had a disseminated viral infection based on symptomology and laboratory values. However, her elevated ferritin level with worsening organ failure with hepatic involvement led us to favor the diagnosis of HLH and initiate immune modulation. In cases of MODS due to HLH, emapalumab may be an attractive alternative to etoposide, which has a wide range of toxicities including the risk of treatment-related myelodysplastic syndrome or acute myeloid leukemia. Further, optimal etoposide dosing can be challenging in an infant with renal and hepatic dysfunction. Emapalumab has a minimal side effect profile compared with etoposide due to its specificity to interferon gamma. Given the similarity of the presentation of this patient to another case in which the CXCL9 level was measured at 39,000 pg/ml (4), we chose to administer a moderate/high starting dose of emapalumab (6 mg/kg). Furthermore, emapalumab pharmacokinetic clearance is enhanced with higher levels of IFN-gamma (8). Because of the clinical efficacy of emapalumab, paired with its minimal side effect profile (9), this is likely an acceptable approach.

It has well been established that the HLH-2004 criteria lack specificity (6). Specificity may be increased by raising the ferritin threshold from 500 to 10,000 ng/ml (10). However, the fact that this patient, with proven Type 2 familial HLH, did not fulfill the HLH-2004 diagnostic criteria until HD#3 (Table 1) also speaks to the lack of sensitivity, or better, the lack of real-time clinical usefulness, of the HLH-2004 criteria. This is due to the specialized immunologic testing needed to fulfill many of the criteria. Lin et al. (7) recently published proteomic-based approach to differentiate HLH from sepsis using the CXCL9/IL-6 ratio. They reported that a CXCL9/IL-6 ratio of approximately 4:1 differentiated HLH from sepsis. Interestingly, the patient’s in-house clinical IL-6 level on HD#4 was measured at 444 pg/ml. Simultaneously, a CXCL9 level was submitted to CCHMC and was ultimately measured at 108,813 pg/ml. Therefore, our patient had a CXCL9/IL-6 ratio of 245:1. In addition, IL-18 has been used to differentiate HLH from sJIA/MAS (11). The IL-18 level of our patient at12,941 pg/ml is significantly elevated, but it remains 10-fold lower than that of patients diagnosed with true sJIA/MAS (unpublished data). These data indicate that there is both a need and an opportunity to improve the HLH-2004 diagnostic criteria.

**Table 1:** Patient fulfillment of HLH-2004 diagnostic criteria.

	Hospital day
Fever	1
Splenomegaly	3
Cytopenias (affecting 2 of 3 lineages in the peripheral blood)	3
Hypertriglyceridemia (>265 mg/dl) and/or hypofibrinogenemia (<150 mg/dl)	3
Hemophagocytosis in bone marrow or spleen or lymph nodes	11
Low or absent NK-cell activity	14
Ferritin 500 mg/L	3
Soluble CD25 (i.e., soluble IL-2 receptor) 2,400 U/ml	9

## Data Availability

The original contributions presented in the study are included in the article/Supplementary Material, further inquiries can be directed to the corresponding author.
